# Troubled sleep: A response to commentaries

**DOI:** 10.1093/emph/eou011

**Published:** 2014-04-08

**Authors:** David Haig

**Affiliations:** Department of Organismic and Evolutionary Biology, Harvard University, 26 Oxford Street, Cambridge, MA 02138, USA

**Keywords:** attachment, strange situation, interbirth interval, infant sleep, mismatch, evolutionary pediatrics

I am flattered by the thoughtful commentaries on my paper. ‘Troubled sleep’ had two major purposes. The first was to draw attention to the oppositely perturbed sleep of infants with Prader–Willi syndrome (PWS) and Angelman syndrome (AS) and explore its evolutionary implications. The involvement of imprinted genes suggests that infant sleep has been subject to antagonistic selection on genes of maternal and paternal origin with genes of maternal origin favoring less disrupted sleep. McKenna [1] is uncomfortable with the notion that night waking depends on a single gene and believes that I discount a significant role for culture. I did not intend to imply that the control of infant sleep was simple. Its pattern will be determined by complex interactions among genes, by cultural practices and by negotiations between individual caregivers and infants.

My second major purpose was a critique of the idea that children would be happier, healthier and better-adjusted if we could only return to natural methods of child care. This way of thinking is often accompanied by a belief that modern practices put children at risk of irrevocable harm. The truth of such claims is ultimately an empirical question, but the claims are sometimes presented as if they had the imprimatur of evolutionary biology. This appeal to scientific authority often seems to misrepresent what evolutionary theory predicts: that which evolves is not necessarily that which is healthy. McKenna’s theoretical stance is, of course, more nuanced than a simple equation of the natural with the good, and I am perhaps guilty of using him as a straw-man by taking published statements out of context.

McKenna and colleagues have done valuable research on how mothers and infants interact while sleeping together and on variation among mother–infant dyads. Their proposal that co-sleeping and night-nursing have important benefits for infants is a reasonable hypothesis and deserves investigation. Evolutionary theory should be informed by observation, but observations are interpreted in terms of implicit or explicit theoretical models and may be misinterpreted if models are faulty.

A single observation is often interpreted differently by different models. Hinde [2], for example, cautions against interpreting night-nursing as a tactic to increase interbirth intervals (IBIs) because marmoset mothers are most frequently woken by their infants around the time that mothers undergo post-partum ovulation [3]. Therefore, she reasons, night-nursing does not inhibit ovulation. However, maximal waking as mothers return to fertility is precisely what would be expected from a model of parent–offspring conflict. The observation does not discriminate between models of parent–offspring harmony or conflict. The return to fertility of tamarin mothers is delayed by more intense suckling [4].

## COSTS OF CONFLICT

Wilkins [5] argues that costs to individual health are an inevitable consequence of evolutionary conflict because systems in which different agents have different agendas are inherently unstable and because genes have pleiotropic effects. If a conflictual system is to evolve to a semi-stable state, then there must be side-costs to each of the parties to restrain futile evolutionary cycles of move and countermove.

Most physiological systems function year after year without significant disease. By contrast, the brief 9 months of pregnancy are characterized by frequent complications for mothers and fetuses even though a favorable outcome of gestation is central to the fitness of both (‘fitness’ here and henceforth refers to genetic rather than physical fitness). Why should pregnancy not be more efficient and more robust than other physiological systems, rather than less? This ‘paradox’ is resolved by the simple observation that natural selection can act at cross-purposes on genes in different bodies but acts toward common goals on genes within genetically uniform bodies. Pregnancy is homeostatically unstable because signaling between mothers and offspring is evolutionarily unreliable [6]. Crucial checks, balances and feedback controls are lacking in the shared physiology of the maternal–fetal unit [7]. Infant sleep may similarly lack the exquisite organization of systems without evolutionary conflict.

We love our babies but are sometimes at a loss to know what they want. Postnatal development, like prenatal development, is subject to difficulties of evolutionarily credible communication between mothers and offspring. Crying ‘can become a trigger for a frustrated parent or caregiver to shake a child’ [8]. Deterioration of maternal psychomotor vigilance because of fragmented sleep may increase risks of accident for both mother and child [9].

Imprinted genes from the PWS/AS gene cluster appear to play a role in arousal from sleep. Infants with PWS have a high frequency of central apneas and a shorter than normal latency from sleep onset until the first episode of rapid-eye movement (REM) sleep [10, 11]. Older individuals exhibit generalized hypoarousal [12, 13] and reduced responses to hypoxia and hypercapnia during non-REM (NREM) sleep [14, 15]. Sleep architecture has not been studied in infants with AS although their general wakefulness is clearly attested. Older children with AS exhibit increased time awake during the night and a reduced proportion of REM sleep [16].

McNamara [17] proposes that paternally expressed imprinted genes (PEGs) should favor REM sleep whereas maternally expressed imprinted genes (MEGs) should favor NREM sleep. Whether sleep structure in PWS and AS supports this hypothesis is unclear (see above). However, in mice, inactivation of the maternal copy of *Ube3a* (a MEG from the PWS/AS region) results in reduced slow wave sleep (a component of NREM) [18] and reactivation of silent paternal copies of *Gnas* increases NREM and decreases REM [19]. Both observations are consistent with McNamara’s hypothesis.

## ATTACHMENT

McNamara [17] draws attention to associations of infant sleep with attachment. Sleep in the first 6 months predicts the pattern of attachment of 1-year-olds assessed in the Strange Situation Procedure (SSP) [20, 21]. Thus, behavior in the SSP has antecedents that are partially expressed in earlier patterns of maternal and infant waking. In particular, infants with an insecure-resistant pattern of attachment (Group C) wake more often at night, and infants with an insecure-avoidant pattern (Group A) less often, than securely attached infants (Group B) [20–22]. These associations are present when waking is assessed by sleep diaries but not by actigraphy [23]. Actigraphy records all arousals whereas sleep diaries record an awakening only if the mother also wakes. Sleep diaries therefore record the category of awakenings that are of particular relevance for the Blurton Jones–da Costa hypothesis.

Infants are classified as having insecure-resistant attachment if they maintain close proximity to their mother after a brief separation in the SSP while expressing negative emotions and exhibiting contradictory behaviors that seem to both encourage and resist interaction. By contrast, infants are classified as having insecure-avoidant attachment if they do not express negative emotion and avoid contact with their mother after reunion [24]. Such an infant ‘appears to many—including experienced developmental psychologists—as a robust, friendly, independent child. It is only when one is reminded that this is an unusual way for a 1-year-old to behave in separation and reunion episodes in a strange environment … that one is inclined to take avoidance seriously’ [24, p. 320]. Insecure, independent, or both? I am not competent to judge. A sharp decrease in night wakings at 7–8 weeks was observed in a group of infants diagnosed with insecure-avoidant attachment at 12 months [21].

Insecure-avoidant and insecure-resistant behaviors might be considered antithetic accommodations of infants to less responsive mothers; the former associated with reduced demands on maternal attention, the latter with increased demands. A parallel pattern is seen in effects on maternal sleep. Insecure-avoidant infants wake their mothers less frequently, and insecure-resistant infants more frequently, than securely attached infants. The insecure-avoidant pattern seems more aligned with the predicted effects of MEGs and the insecure-resistant pattern with the predicted effects of PEGs.

One way that infants engage their mothers’ attention is by smiling and laughing. Maternal attention becomes focused on the child and distracted from other activities. Infants with PWS have weak cries and flat affect [25] whereas those with AS are noted for frequent smiles and laughter [26]. The positive affect of AS has been interpreted as an adaptation of PEGs for eliciting maternal investment [27, 28]. Smiles of children with AS attract more adult attention than smiles of children with other intellectual disabilities [29] and decline in frequency with age [30].

Parent–child interactions are transformed once children can speak. Wilkins [5] suggests that sleep fragmentation is correlated with delayed language development. He cites a twin study in which sleep consolidation at 6 months was highly heritable and genetically correlated with language skills at 18 and 30 months. Specifically, infants with more fragmented sleep at 6 months had less language at 18 and 30 months [31]. Infants with AS have unconsolidated sleep and never learn to speak [32]. The absence of language in the absence of expression of one or more MEGs is compatible with a hypothesis in which earlier development of language reduces infant demands on mothers. A burgeoning literature attempts to explicate the relation between genomic imprinting and acquisition of human language [33–35].

## CULTURE

Hinde [2] contrasts the sleep expectations of WEIRD (Western, educated, industrialized, rich, democratic) parents with patterns of infant sleep in the ARE (adaptively relevant environment) and McKenna [1] remarks ‘it is no surprise that western parents … remain the most obsessed, judgmental, disappointed, exhausted, and the least satisfied parents on the planet!’ What might be found, he asks, in cultures with different assumptions and expectations about infant sleep such as China, Japan, Vietnam, India or the Philippines. All five of these countries were included in a cross-cultural survey of parentally reported sleep problems in infants and toddlers [36]. Table 1 summarizes data for the five countries mentioned by McKenna and the five ‘western’ countries in the survey. Sleep problems were reported in all countries by some parents, albeit at substantially different frequencies. The western countries had distinctive sleeping arrangements but were not outliers on parental perceptions of problematic sleep.

**Table 1.** Percentage of parents reporting a sleep problem compared with the percentages of children sleeping in their own room or in the parental bed (data from [36])

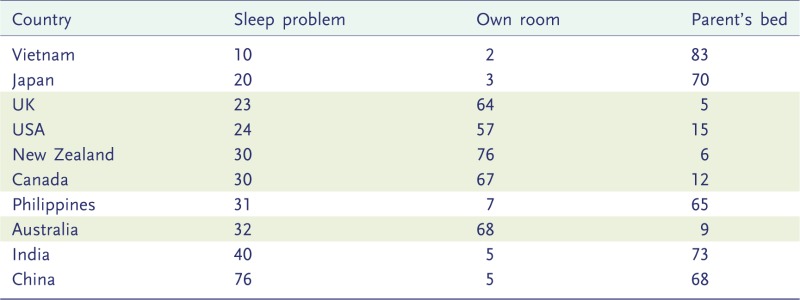

The populations are ordered from lowest to highest reported rates of sleep problems. The five, predominantly anglophone, ‘western’ countries are shaded. The questionnaires were completed online in all countries except Vietnam where they were completed in face-to-face interviews.

Japan has high rates of bed-sharing and low rates of problematic sleep [37]. Nevertheless, when space is available, some Japanese parents choose western sleeping arrangements and a market exists for Japanese-language books on how babies can be trained to sleep through the night (M. Wada, personal communication). China, Taiwan and Hong Kong have both high rates of bed-sharing and high rates of problematic sleep compared with western countries. Within this grouping, however, more children sleep in their own room but parents report fewer sleep problems in Hong Kong than in either China or Taiwan [36]. Clearly, cultural differences are significant, and the causes of this variation should be investigated, but the differences cannot be summarized simply as ‘west is worst’.

The fitness gain to mothers of an extra child and the benefits for infants of longer IBIs are substantial. These selective forces are unlikely to be orders of magnitude weaker than the advantages of lactase persistence, yet the selective forces associated with dairying have been sufficient to result in adaptive genetic differentiation among populations [38]. The possibility of gene–culture coevolution should not be discounted for behaviors associated with infant-care practices.

## MISMATCH

McKenna [1] and Hinde [2] both emphasize benefits from night-nursing other than prolongation of IBIs, including the nutritional value of milk for infants. I do not deny these benefits. The net effect of natural selection will be determined by the aggregate of all fitness-related effects. Genes in infants will be selected to favor more frequent night waking for both nutritional and contraceptive reasons.

Mismatch between modern and ancestral environments can be a cause of disease, but I remain skeptical of a tendency to ascribe most modern woes to incongruence between our evolved nature and western cultural practices. We did not evolve to be happy or healthy but to leave genetic descendants, and an undue emphasis on mismatch risks conflating health and fitness.

McKenna [1] writes ‘It isn’t really nice nor maybe even possible to fool mother nature’. Here I disagree. Our genetic adaptations often try to fool us into doing things that enhance fitness at costs to our happiness. When tempted by instincts, we should be wary of false advertising and genetic strategies of bait and switch. Fitness-enhancing behaviors are often elicited by the promise of happiness but fail to deliver as promised. Our genes do not care about us and we should have no compunction about fooling them to deliver benefits without serving their ends. Contraception, to take one obvious example, allows those who choose childlessness to enjoy the pleasures of sexual activity without the fitness-enhancing risk of conception.

Night waking evolved in environments in which there were strong fitness costs from short IBIs and in which parents lacked artificial means of birth-spacing. If night waking evolved because it prolonged IBIs, then it may no longer serve the ends for which it evolved or, at least, these ends have been greatly attenuated. Nevertheless, optimal infant development might continue to depend on frequent night feeds as part of our ingrained evolutionary heritage. It could also be argued that when night waking is not reinforced by feeding, and infants sleep through the night, then conflict within their genomes subsides. Infants would then gain the benefit of unfragmented sleep without the pleiotropic costs of intragenomic conflict. Plausible arguments could be presented for either hypothesis and a choice between them must await discriminating evidence.

Whether particular environmental mismatches are causes of ill-health remains an open question. Hunter-gatherer babies were born on walkabout, sleeping out on cold nights next to warm bodies, but obstetrical wards are maintained within a narrow range of lukewarm thermoneutrality in the belief that modern babies should be neither too hot nor too cold. The tightly wrapped baby is then brought home to a centrally heated, air-conditioned house that is unlike any environment experienced by its infant ancestors. Does this mismatch matter? Are there developmental consequences of experiencing a narrow range of temperatures during infancy? The proportion of active sweat glands is modified by temperatures experienced during the first 2 years of life but then remains unchanged by subsequent migration to hotter or colder climates [39]. Could recruitment of brown adipose tissue be similarly compromised in the absence of early cold exposure with long-term consequences for energy expenditure? These are interesting questions for research but it would be reckless to recommend saunas and cold-hardening for infants without clear evidence of benefit and absence of harm.

## THE HARMONY OF NATURE

Parents and offspring are distinct individuals. What is best for one need not be best for the other whether ‘best’ is defined with respect to health or fitness. My focus has been on conflicts between genes expressed in mothers and offspring, but intergenerational dilemmas can also arise in medicine and public health, whether these are clinical predicaments in which the treatment that minimizes risk to the mother does not minimize risk to the child [40], ethical conflicts pitting respect for maternal autonomy against beneficence toward a soon-to-be child [41], or competition for funding between advocates of maternal and child health [42].

Crespi [43] notes that concepts of parent–offspring conflict have been neglected by academic and clinical medicine, but similar neglect occurs in evolutionary biology. Most discussions of the evolution of human life history do not consider kin conflicts and assume that natural selection acts to maximize individual fitness. For example, the ‘obstetrical dilemma’ posed by the tight fit of the infant head to the maternal pelvis has consequences for both maternal and infant fitness, but its origin is usually not discussed in terms of the distinct interests of mothers and offspring (but see [44]).

Humans seem strongly predisposed toward viewing parent–offspring relations as fundamentally harmonious. In my experience, conflicts of interest between parents and offspring are readily conceded by offspring but less readily by parents. Parents recognize that their child sometimes sees their relations as conflictual but nevertheless believe that they act in the child’s best interests because they better understand what is good for the child. This parental justification is sometimes defensible, but sometimes contains an element of self-serving rationalization. The question of what a parent would accept in exchange for a child’s life is abhorrent, but parents often make decisions that balance their child’s needs—attending a school recital or help with homework—against other demands on their time.

Our views of family life are shaped by potent myths. The archetypal image of Madonna and child is emotively powerful. Idealizations of parenthood, at least in the abstract, are an expression of a broader predisposition to view nature as fundamentally beneficent. But, if the natural is good, then disease must result from some ‘unnatural’ disturbance of nature’s balance. In the creation myth of Genesis, women suffer pain in childbirth as punishment for eating the forbidden fruit. When our lives do not match our ideal vision of how things ought to be we tend to blame ourselves rather than the vision. We see modern ills as the fruits of our fall from grace. Together with Crespi [43], I believe that a more realistic evolutionary view, in which conflicts and ambivalence are seen as an inescapable part of family life, would be good for our emotional health.

**Conflict of interest**: None declared.
